# Current Concepts on Gastric Carcinoid Tumors

**DOI:** 10.1155/2012/287825

**Published:** 2012-12-17

**Authors:** George C. Nikou, Theodoros P. Angelopoulos

**Affiliations:** Section of Gastrointestinal Neuroendocrinology, First Department of Propaedeutic Internal Medicine, Laiko Hospital, University of Athens Medical School, Agiou Thoma 17, Goudi, 11527 Athens, Greece

## Abstract

Gastric carcinoid tumors (GCs) are rare lesions representing less than 10% of carcinoid tumors and less than 1% of all stomach neoplasms. There are three distinct types of gastric carcinoids; type I includes the vast majority (70–85%) of these neoplasms that are closely linked to chronic atrophic gastritis. Type II which accounts for 5–10 %, is associated with Zollinger-Ellison syndrome and often occurs in the context of multiple endocrine neoplasia type 1. Type III, finally, represents 15–25% of gastric carcinoids and is characterized by a far more aggressive course. The optimal clinical approach to GCs remains to be elucidated, depending upon type, size, and number of carcinoids. While there is universal agreement about the surgical treatment of type III GCs, current options for type I and II include simple surveillance, endoscopic polypectomy, surgical excision associated with or without surgical antrectomy, or total gastrectomy. Moreover, the introduction of somatostatin analogues could represent another therapeutic option.

## 1. Introduction

Neuroendocrine tumors (NETs) are tumors of the interface between the endocrine and nervous system. They are characterized by the presence of secretory granules as well as the ability to produce biogenic amines and polypeptide hormones. These tumors originate from endocrine glands such as the adrenal medulla, the pituitary, and the parathyroids, as well as endocrine islets within the thyroid or the pancreas, and dispersed endocrine cells in the respiratory and gastrointestinal tract. The clinical behaviour of NETs is extremely variable; they may be functioning or nonfunctioning, ranging from very slow-growing tumors, which are the majority, to highly aggressive and very malignant tumors. 

The term gastroenteropancreatic neuroendocrine tumors (GEP NETs) has prevailed and includes both gastrointestinal (GI) neuroendocrine tumors/carcinoids and pancreatic endocrine tumors (PETs). They are thought to arise from local gastrointestinal stem totipotent cells, rather than from the neural crest, as assumed at first [1]. According to the histological classification of the tumors developed by the World Health Organization (WHO) in 2010 (Table 1), GEP NETs are classified as the following:

- well-differentiated neuroendocrine neoplasms low- and -intermediate grade (G1, G2);
- poorly differentiated neuroendocrine neoplasms-high grade (G3) [2].

Two debated terminological issues have arisen with novel classifications. The use of the termendocrineversus neuroendocrine and that of neoplasms instead of tumors (neuroendocrine neoplasms—NEN). In addition, well-differentiated (low- and -intermediate grade) gastrointestinal NETs have been variably termedcarcinoid tumors. Although there may be arguments favoring each term, it must be recognized that they are essentially synonymous and are widely understood. For the sake of uniformity,neuroendocrinetumors and gastric carcinoid tumors will be used throughout this paper. Classifications based on the TNM system and taking also age and depth of invasion into account have been providing patients and clinicians with meaningful prognostic information, but are used secondarily [3, 4].

Gastric carcinoid tumors (GCs) are relatively rare lesions representing about 7% of all carcinoid tumors and less than 1% of all stomach neoplasms. However, there are reports which suggest that these lesions may actually be far more common. There are three subtypes of GCs each one with a distinct pathophysiologic mechanism, resulting in diverse clinical outcomes and demanding different management [5–7] (Table 2). Type I gastric carcinoids (GC-I) (approximately 70–80% of the total) are associated with autoimmune chronic atrophic gastritis. They are more common in women [8]. Complete oxyntic mucosal atrophy results in achlorhydria and intrinsic factor deficiency. In response to persistent achlorhydria, G cells in the gastric antrum undergo hyperplasia and secrete more gastrin resulting in hypergastrinemia. Approximately, 5% of patients with autoimmune chronic atrophic gastritis will develop a gastric carcinoid tumour [9, 10]. These tumors have a good prognosis, with 5-year survival quoted at 96% that does not differ from an age-matched normal population [11].

Type II lesions are associated with gastrinomas resulting in Zollinger-Ellison syndrome (ZES). Patients' hypergastrinemia does not result from parietal cell loss, but is due to gastrin secreting G cell neoplasia in association with ZES and/or multiple endocrine neoplasia type 1 (MEN-1). They account for approximately 5–8% of gastric carcinoids [12]. The majority of GC-II have a good prognosis, but a minority behave more aggressively and up to 30% of them metastasize. They present in up to 20% of patients with ZES and MEN-1. Whilst type I lesions are limited to the mucosa of the gastric body and fundus, type II lesions have occasionally been described in the antrum [13, 14].

Type III is a sporadic disease associated with normal gastrin levels; it has the highest rate of metastases (>50%), thus the worst prognosis [15]. Unlike the other two types of gastric carcinoids, it has been shown to have a higher frequency in men. It presents with solitary, ulcerated, and deeply invasive lesions, usually larger than 1-2 cm. It may also be associated with the presentation of an atypical form of carcinoid syndrome where itching, cutaneous wheals and bronchospasm predominate, due to the high levels of histamine released from enterochromaffin-like (ECL) cells.

 Another type of GCs has been described and is classified as type IV. This extremely rare type is derived from different endocrine cells of the stomach, such as those producing serotonin or gastrin and may have a very aggressive course [16].

## 2. Epidemiology

 A recent marked increase in GCs incidence has been noted that to be attributed to some of the following reasons: the wide use of upper endoscopy as a screening tool, the periodical gastroscopies of the same person, the routine habit to obtain biopsies in the course of upper gastrointestinal endoscopy, the application of specific immunohistological identification techniques, and the greater clinical focus on the subject [17, 18]. The widespread use of proton pump inhibitors can also induce gastric achlorhydria, thus contributing to hypergastrinemia. Moreover, the importance of genetic and molecular background remains to be elucidated.

Gastric carcinoids account for 0.6–2% of gastric polyps excised [19]. However, the number of gastric carcinoids reported is increasing from 0.3 cases per million in 1981 to 1.8 cases in 2000 and more than 2.5 cases per million in 2010. Whether this is a genuine increase in incidence or simply a reporting artefact is not currently clear. There also appears to be a dynamic change in gender distribution, as more than two-third of gastric carcinoids reported in 2010 were in women (compared with 51.6% of all gastrointestinal carcinoids), whereas in 1970 only 55% were reported in women [20, 21]. This may again reflect an increasing number of type I lesions (which are more common in women) now being discovered serendipitously by endoscopy, whereas in the past there was an increased proportion of symptomatic type III tumors, which occur more commonly in men.

## 3. Pathophysiology

**Figure 1:**
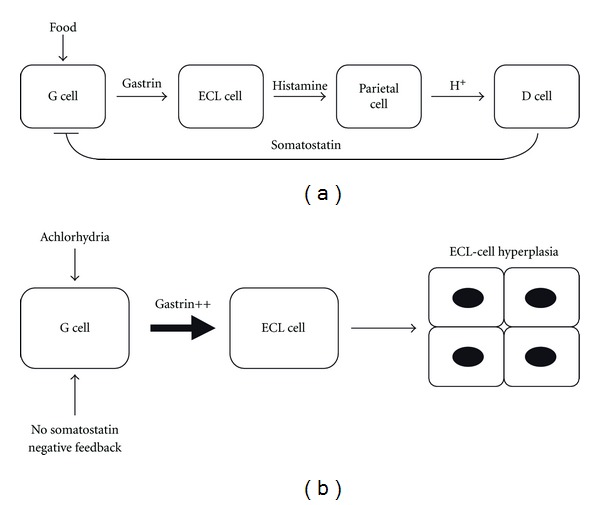
Pathophysiologic mechanisms of normal acid secretion from parietal cells after a meal (a) and ECL-cell hyperplasia in patients with achlorhydria and loss of somatostatin negative feedback (b).

 Types I and II gastric carcinoid tumors are associated with hypergastrinemia. In response to food, gastrin is secreted by antral G cells and binds predominantly to cholecystokinin (CCK) receptors located on the ECL-cell membrane thereby triggering histamine release. Histamine subsequently binds to H2 receptors located on parietal cells, thus stimulating acid secretion (Figure 1(a)) [22]. In addition to its acid secretagogue properties, gastrin stimulates gastric epithelial cell proliferation, but as the proliferating and putative stem cells in the stomach do not express CCK-2 receptors, this is thought to be secondary to the release of other growth factors such as heparin-binding epidermal growth factor and transforming growth factor a [23, 24]. During the development of gastric carcinoids, however, gastrin appears to exert direct pro-proliferative effects upon ECL-cells (Figure 1(b)). In autoimmune chronic atrophic gastritis, gastric parietal cells are unable to secrete acid and the consequent achlorhydria results in G cell hyperplasia and hypergastrinemia. Gastrin exerts trophic effects upon ECL-cells, which undergo hyperplasia and in some cases progression to GC-I occurs. As only a minority of patients with autoimmune chronic atrophic gastritis, achlorhydria, and hypergastrinemia develop gastric carcinoid tumors, factors in addition to gastrin are required for tumor development. Other conditions that result in hypergastrinemia such as vagotomy and chronic proton pump inhibitor (PPI) use are not associated with the development of gastric carcinoids in humans. This suggests that although hypergastrinemia is an essential prerequisite for the development of type I and II tumors, it on its own is not sufficient for tumor formation [25].

**Figure 2:**
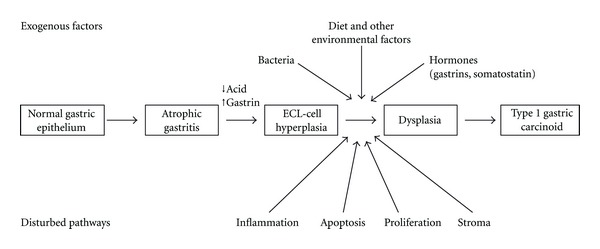
Factors contributing to type 1 GCs development.

A number of cofactors for the development of gastric carcinoid tumors have therefore been proposed. These include genetic mutations, growth factors, bacterial infection and effects on the underlying mesenchyme (Figure 2). These factors may affect a number of cellular pathways such as apoptosis, autophagy, proliferation, and differentiation in order to promote tumor development. Loss of heterozygosity at MEN-1 gene locus 11q13 has been found in all GC-II, in 17–73% of GC-I and in 25–50% of GC-III, even though these tumors do not develop in MEN-1 patients [26]. A role for the apoptosis-inhibiting protein BCL-2 has also been proposed, with the hypothesis that the antiapoptotic activity of BCL-2 may contribute to the development of carcinoid tumors by extending the exposure of hyperplastic ECL cells to other, so far unknown, oncogenic factors. Mcl-1 protein expression is also increased specifically in human hypergastrinemia-associated GC-I. Gastrin-induced mcl-1 expression may therefore be an important mechanism that contributes toward type I GCs development [27, 28].

## 4. Diagnosis

### 4.1. Clinical Features

**Figure 3:**
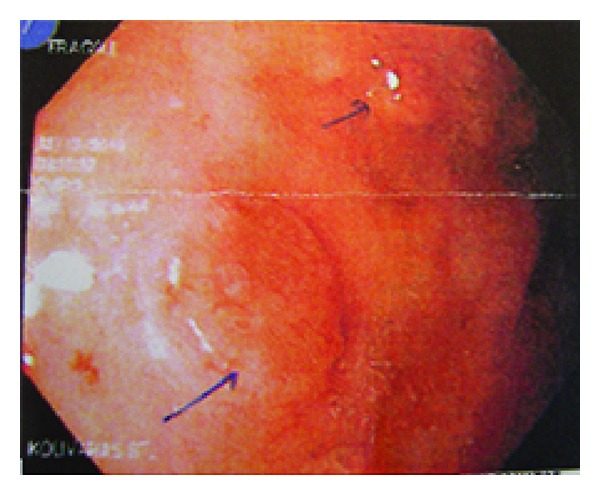
Macroscopic appearance of type 1 gastric carcinoid tumors during gastroscopy.

The clinical presentation of gastric carcinoids is often nonspecific, and many lesions are detected at routine endoscopy as incidental findings. In a series of 65 patients with gastric carcinoids, 19 (29%) were diagnosed by screening of patients with pernicious anaemia; these patients were asymptomatic and by definition had type I lesions [29]. Gastric carcinoids usually have the endoscopic appearance of mucosal polyps (Figure 3). In type I and II diseases, several polyps are often seen in clusters, whilst type III lesions are usually solitary. The surrounding mucosa may be macroscopically normal, especially in type III lesions, or there may be evidence of atrophy (type I) or associated peptic ulcer (type II). The distribution of lesions is predominantly in the gastric body, although microscopic type II lesions have been described in the antrum and sporadic lesions may occur anywhere in the stomach. Histological analysis of a lesion is the definitive diagnostic tool; however, it is also useful to analyze biopsies taken from apparently unaffected mucosa in order to identify possible background conditions, such as atrophic gastritis and to assess for the presence or absence of microcarcinoids [30]. 

In addition to the incidental presentation, a subgroup of gastric carcinoids will cause symptoms. These symptoms may either result from local mechanical effects or have a neuroendocrine basis. Some patients therefore present with abdominal pain, nausea, or gastrointestinal hemorrhage because of the local effects of the tumor. A number of type III tumors have been shown to be associated with concomitant vascular abnormalities and to present with severe hemorrhage [31]. It has been proposed that this may occur as a result of angiogenic field effects related to tumor production of growth factors. In rare instances, the clinical features of carcinoid syndrome of cutaneous flushing and diarrhea have been described. However, whereas the “classical” carcinoid syndrome associated with midgut and hindgut carcinoids is mediated by 5-hydroxytryptamine (serotonin), the syndrome associated with ECL-cell tumors is “atypical” and is associated with 5-hydroxytryptophan, because of deficiency of the enzyme dopa-decarboxylase responsible for conversion of 5-hydroxytryptophan to 5-hydroxytryptamine. The atypical syndrome is associated with more intense and protracted purplish flushing; the limbs as well as the upper trunk are often affected and telangiectasias are commonly observed [32].

### 4.2. Laboratory Findings

Serum chromogranin-A (Cg-A) appears to be the most useful diagnostic marker in the diagnosis of gastric carcinoids, as well as the rest of the neuroendocrine tumors. It has a sensitivity higher than 90% in many studies (86% for chromogranin-B and 5% for chromogranin-C) and is well correlated to tumor burden, especially in the presence of liver metastasis, making it a valuable marker in followup after treatment is initiated [33, 34]. It is much more specific than gastrin in cases of type I GCs where hypergastrinemia due to atrophic gastritis occurs (55–85% versus 35–55%) [35]. Patients with ZES also show significantly elevated levels of Cg-A as a result of the trophic action of gastrin on the endocrine cells of the gastric mucosa, which leads to its secretion [36]. Correlation with type III GCs is not so well established. 

The overall specificity of Cg-A ranges between 50–87% in various studies, depending on cut-off values. High values of Cg-A can be found not only in other neoplasms, but also in benign conditions (renal failure, liver failure, atrophic gastritis, and inflammatory bowel disease) [37].

 Type I GCs are related to hypochlorhydria and atrophic gastritis, as mentioned above. In patients with this condition, a full blood count and levels of vitamin B12 are useful initial investigations. If pernicious anemia is suspected, measurement of antibody levels against parietal cells and intrinsic factor should take place. Physicians should also bear in mind that pernicious anemia is an autoimmune disease related with other conditions with an autoimmune mechanism, like diabetes, Hashimoto thyroiditis and primary biliary cirrhosis. 

 Type II GCs are presented in the context of hypergastrinemia and the Zollinger-Ellison syndrome. Serum gastrin and gastric pH levels are indicative of the diagnosis. If gastrin is >1000 ng/mL (normal value <90 ng/mL) and gastric fluid's pH is <2, ZES is certain. If gastrin is 100–1000 ng/mL and pH < 2, then gastrinoma is possible in the right clinical context. Secretin and/or protein meal test should follow to establish the diagnosis [38]. MEN-1 is present in about one fourth of the patients with ZES, thus levels of PTH, Ca, P, and prolactin should be measured, followed by a CT/MRI scan of the pituitary gland and molecular screening for mutations in the MENIN gene, if applicable. Proton pump inhibitors interfere with gastrin secretion and should be withheld at least two weeks before blood tests.

### 4.3. Imaging Modalities

Abdominal ultrasound and CT/MRI scans are useful when metastatic disease is present. Endoscopic ultrasonography (EUS) can provide useful information in large tumors >1 cm regarding the exact depth of tumor invasion and positive tumor margins after endoscopic removal [39]. Somatostatin receptor scintigraphy (Octreoscan) has been used since the early 1990s as a means of localizing both primary and metastatic tumors expressing somatostatin receptors. Unfortunately, Octreoscan is often negative in early type I and II GC's making it of limited use, mostly in detecting metastatic disease [40, 41]. Standard 18F-fluorodeoxyglucose positron emission tomography (PET) is also of limited value when assessing neuroendocrine tumors. However, 11C-5-hydroxytryptophan and 6-[18]fluoro-L-dihydroxyphenylalanine (18F-dopa), among others, may be more useful PET tracers in these tumors. Nevertheless, PET's role in gastric carcinoids is unclear and not supported by literature [42, 43]. 

### 4.4. Histopathology

The diagnostic accuracy and correct characterization of GCs require not only removal and biopsy of the largest polyps, but also extensive sampling from both the antrum (two samples) and the body/fundus (four samples). Histochemical assessment of chromogranin-A and synaptophysin is very important in identifying hyperplasia, dysplasia, and malignant transformation of ECL cells. Apart from that, immunohistochemical determination of the proliferative index Ki-67 and evaluation of the mitotic index, by counting number of mitoses per 10 high-power fields (HPFs), is mandatory for a more accurate management plan. 

## 5. Treatment

The clinical approach to GCs is largely dependent upon the type and size of the lesions. Management of GC-III is fairly clear and comparable to that used for gastric adenocarcinomas, which includes partial or total gastrectomy with extended lymph node resection. 

**Figure 4:**
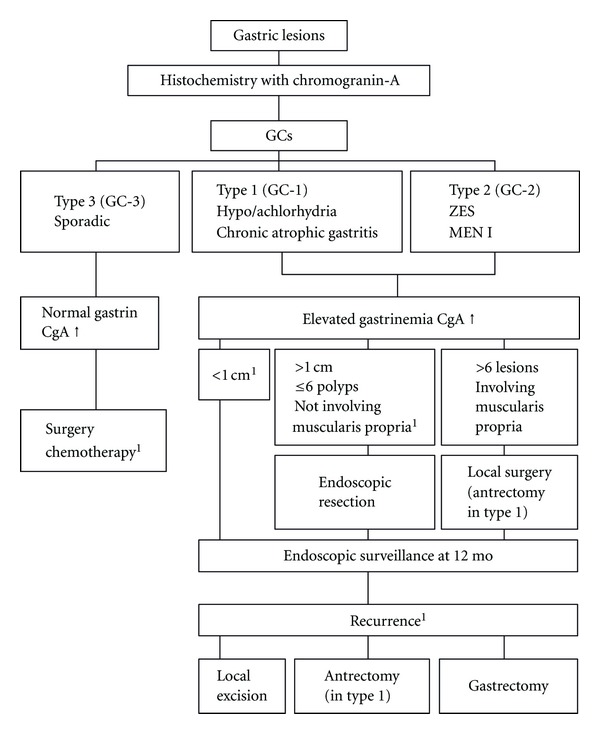
Management flow chart of GCs according to ENETS guidelines. ^1^Consider SSAs.

Management of type I and II GCs is more controversial because they are characterized by a more benign biological behaviour. The European Neuroendocrine Tumor Society (ENETS) Consensus Guidelines have recently suggested that annual surveillance is appropriate when dealing with patients with GC-I less than 10 mm in size (Figure 4) [44]. In cases of tumors >10 mm in size and in the presence of up to six polyps not involving the muscularis propria at EUS examination, endoscopic resection remains the preferred approach. In the presence of deep gastric parietal wall invasion and positive margins following endoscopic mucosal resection, surgical resection of the tumor should be carried out [45–47].

 Management approach to GC-II has to be considered in the context of MEN-1 syndrome that is commonly present in these patients. Endoscopic treatment can be an option, whereas gastric surgery should be performed only in highly selected patients, particularly in the presence of a histological examination that shows features of poorly differentiated endocrine tumors. The question of whether or not to recommend duodenal-pancreatic surgery in patients with MEN-1 who have pharmacologically controllable ZES and no other clinically evident hormonal excess syndrome is difficult to answer [48].

 Many case series have recently been published advocating antrectomy as a means of gastrin suppression in type I gastric carcinoids [49, 50]. These studies have demonstrated this to be an effective method of reducing volume of both ECL-cell hyperplasia and gastric carcinoids. Confirmation of this strategy of removal of gastrin secretion as an effective method for treating gastrin dependent lesions is provided by evidence of regression of type II lesions following successful gastrinoma excision. However, it is difficult to predict which tumors are still gastrin-responsive and which have progressed beyond this point and are growing autonomously independent of gastrin.

Over the last few years, somatostatin analogues (SSAs) have been tried in the treatment of patients with either GC-I or GC-II, based on their ability to inhibit gastrin release from antral G cells, thus reducing ECL-cell hyperplasia [51]. The use of SSAs, apart from reducing gastrin hypersecretion, may also exert an antiproliferative effect on the hyperplastic or dysplastic ECL cells and reduce the risk of further lesion development by suppressing intestinal metaplasia. This conservative approach is not associated with the possible complications of an antrectomy, offering the opportunity of medically-induced reduction of the size and number of ECLomas that is commonly preferred by patients with severe dyspeptic symptoms [52]. Nevertheless, somatostatin analogues' cost effectiveness is admonishing and gastric carcinoids tend to recur soon after their cessation [53]. 

Conventional chemotherapy may have some utility in undifferentiated or highly proliferating tumors with a negative Octreoscan [54]. Hepatic metastases, depending on size, location, and number, may be amenable to surgical resection or radiofrequency ablation. If surgery is not feasible, embolization either alone (bland), or in combination with chemotherapeutic agents, or using radioactive microspheres can be used. 

Finally, gastrin receptor antagonists and antibodiesagainstprogastrin-releasing peptide have only recently started being studied as alternative therapeutic options for patients with type I and II GCs. The oral gastrin receptor antagonist netazepide showed some promise when used in eight patients with multiple type I GCs and larger studies are anticipated to find out whether its use could be justified [55, 56]. 

## 6. Prognosis

GCs are generally considered benign conditions and patients with GC-I tumors have a life expectancy comparable to that of the general population. The overall 5-year survival rate for all three types approaches 75%, varying from 100% for locally confined, type I GC's, to 21,2% where metastatic disease is present. Type II GC's have a similar outcome to GC-I, although their overall survival is closely related to the course of the associated gastrinoma, with a 5-year survival of 62%–75%. In type III GC's, the presence and extent of liver metastases plays the main role, as patients have 80% 1-year survival in the presence of a solitary, small lesion, compared to 10–16% in cases with numerous lesions or high metastatic burden [57]. 

 Overall mortality rate is practically 0% for type I, 10% for type II, and 25–30% for GC-III. A cumulative analysis of GC's in the Surveillance, Epidemiology and End Results (SEER) database from 1992 to 2004 has indicated that distant metastases or regional spread were evident in 10%–30% of cases at the time of diagnosis, thus suggesting that the widespread opinion regarding the benign behavior of GC tumors might need to be revised [58].

## 7. Conclusion

The incidence of gastric neuroendocrine neoplasms/carcinoids has increased significantly based on widespread use of endoscopy and a greater pathological awareness of the condition. They are still considered rare tumors, though, and are composed of three categories, each one with distinct pathophysiologic mechanisms and clinical features. The treatment of type I and II tumors depends on their size and invasiveness, whereas type III tumors are poorly differentiated neuroendocrine carcinomas and warrant aggressive surgical resection.

## Figures and Tables

**Table 1:** WHO 2010 classification of NEN/NETs.

	Grade 1	Grade 2	Grade 3*
Metastases	−	−	+
Muscularis propria invasion	−	±	+
Tumor size (cm)	≤2	>2	Any
Mitoses/10 HPF**	<2	2–20	>20
Ki 67 index %	≤2	3–20	>20
Angio-invasion	Never	Late	Always

*Grade 3 are divided into small cell and large cell neoplasms.

**HPF: high-power fields.

**Table 2:** Characteristics of gastric carcinoid tumors.

	Type I	Type II	Type III
Proportion of gastric carcinoids	70%–80%—most common	Less than 5%	15%–20%
Associations	Chronic atrophic gastritis, pernicious anemia	MEN-1, Zollinger-Ellison syndrome	Sporadic carcinoid syndrome
Epidemiology	Typically women 50–70 yrs old	Family history of MEN-1 syndrome	Increased in African Americans, most common in men
Plasma gastrin levels	High	High	Normal
Gastric acid output	Low	High	Normal
Number of tumours	Multiple	Multiple	Single
Size of tumors	<1 cm	<1 cm	2–5 cm
Site of tumors	Fundus	Fundus (occasionally antrum)	Fundus or antrum
Metastasis	2–5%	<10%	>50%
Mean age at diagnosis	63	50	55
Prognosis	Good	Usually good—a minority of tumors are more aggressive	Poor
